# Supplementary material for: Relationship between Food Allergy and Endotoxin Concentration and the Toleration Status at 2 Years: The Japan Environment and Children’s Study
Source: Nutrients. 2023 Feb 15;15(4):968. doi: 10.3390/nu15040968 (PMC9959381; doi:10.3390/nu15040968)
Supplement: Supplementary file 1 [file nutrients-15-00968-s001.zip › nutrients-2178569-supplementary.pdf]

Table S1. Symptoms in children with hen's egg allergy and cow's milk allergy in this study

Symptoms	Hen's egg allergy		Cow's Milk allergy	
	N#	N (%) in the item	N#	N (%) in the item
pruritus and urticaria	170	83.3	56	77.8
Swelling of the face, lips, and others	16	7.8	13	18.1
Pale faces	2	1.0	3	4.2
Clouded consciousness	0	0.0	1	1.4
Cough	6	2.9	7	9.7
Wheeze	5	2.5	5	6.9
Vomiting	37	18.1	16	22.2
Diarrhea	20	9.8	15	20.8
Others	23	11.3	7	9.7

#: Number of children without missing value.
